# Benign multicystic peritoneal mesothelioma: a case report

**DOI:** 10.1186/1752-1947-4-385

**Published:** 2010-11-29

**Authors:** Xanthi Pitta, Efstathios Andreadis, Athanasios Ekonomou, Athanasia Papachristodoulou, Chrisostomos Tziouvaras, Leonidas Papapaulou, Nikolaos Sapidis, Thomas Chrisidis

**Affiliations:** 1 Department of Radiology, General Hospital "Agios Pavlos", Ethn. Antistaseos 161, 55134 Thessaloniki, Greece; 2 Department of General Surgery, General Hospital of Edessa, Terma Egnatias 58200 Edessa, Greece; 3 Euromedic Imaging Diagnostic Center, Thessaloniki, Greece; 4 Laboratory of Pathology, General Hospital of Edessa, Terma Egnatias 58200 Edessa, Greece

## Abstract

**Introduction:**

We report the case of a patient with a benign multicystic peritoneal mesothelioma and describe its appearance on computed tomography scans and ultrasonography, in correlation with gross clinical and pathological findings.

**Case presentation:**

A 72-year-old Caucasian woman presented to our emergency department with acute abdomen signs and symptoms. A clinical examination revealed a painful palpable mass in her left abdomen. Abdominal ultrasonography and computed tomography demonstrated the presence of a large cystic mass in her left upper abdomen, adjacent to her left hemidiaphragm. The lower border of the mass extended to the upper margin of her pelvis. A complete resection of the lesion was performed. Pathological analysis showed a benign multicystic peritoneal mesothelioma.

**Conclusions:**

Benign multicystic peritoneal mesothelioma is a rare lesion with a non-specific appearance on imaging. Its diagnosis always requires pathological analysis.

## Introduction

Benign multicystic peritoneal mesothelioma is an uncommon lesion arising from the peritoneal mesothelium. It is often diffuse and shows a marked predilection for the surfaces of the pelvic viscera [1-8]. In our case report, the lesion was solitary and situated in the left abdomen. This disease is a rare medical entity and there are challenges in determining its origin, pathogenesis, diagnosis and therapy.

## Case presentation

A 72-year-old Caucasian woman was admitted to our surgical department having experienced diffuse abdominal pain and discomfort, nausea and vomiting for the previous two days. Her medical history included diabetes mellitus and arterial hypertension, for which she was on medication. She had no relevant family history and did not smoke or drink alcohol.

On physical examination, she showed signs of acute abdomen and a palpable painful mass in her left abdomen was noted. She was tachycardic and laboratory tests showed a white blood cell count of 13,000 cells per cubic millimeter. Her chest and abdominal radiographs did not reveal any abnormalities.

**Figure 1:**
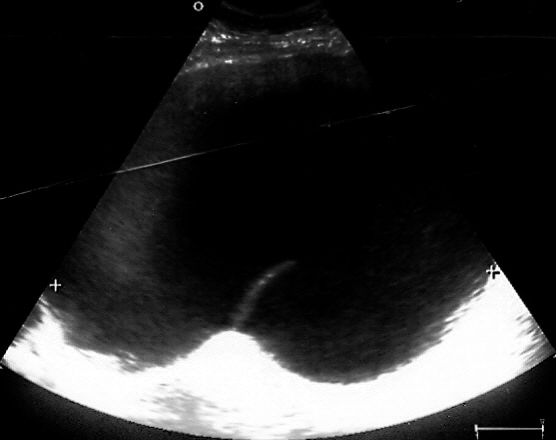
**US image showing a cystic mass with internal septa**.

An ultrasonography (US) examination demonstrated a complex cystic mass with internal septa, without increased vascularity. The source organ could not be identified (Figure 1).

**Figure 2:**
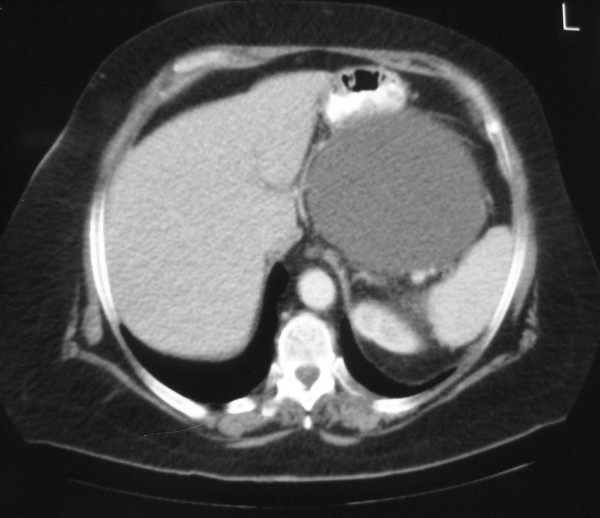
**CT axial image after the intravenous administration of a contrast medium demonstrating an intra-peritoneal hypodense non-enhancing mass adjacent to the stomach and spleen**.

**Figure 3:**
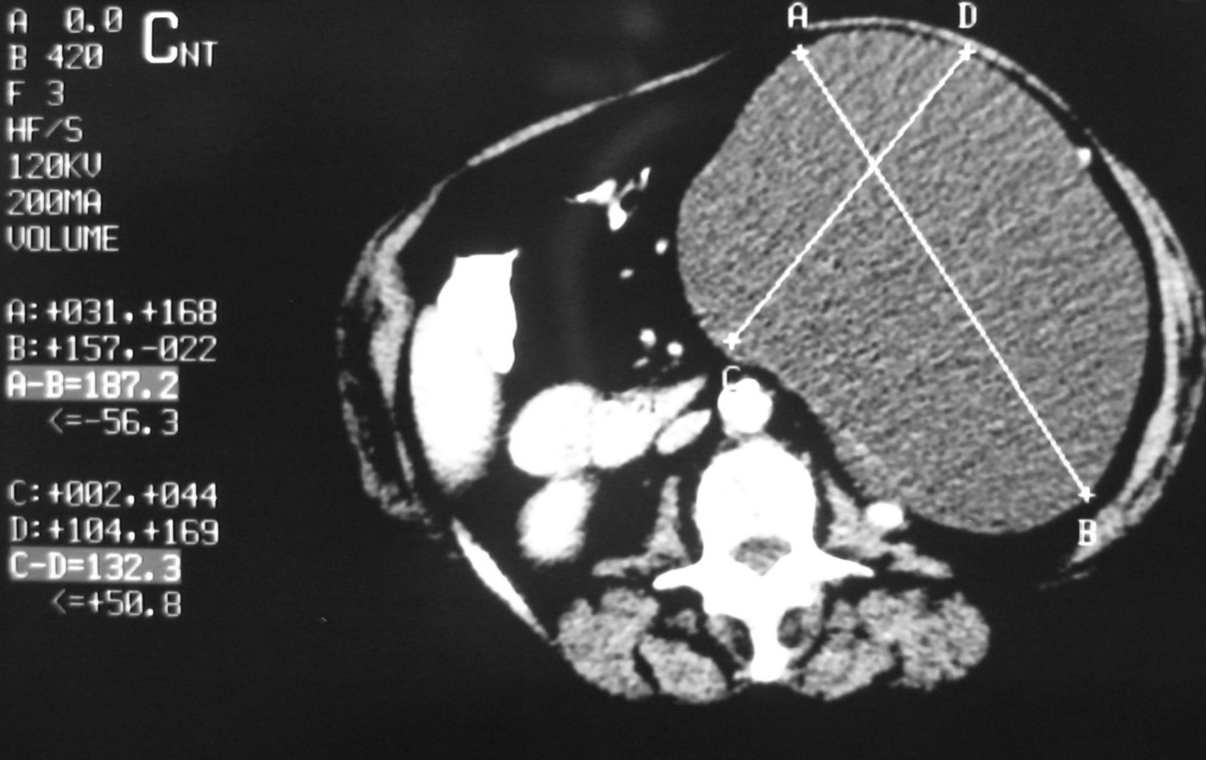
**Demonstration of the maximum dimensions of the mass, on CT axial plane**.

**Figure 4:**
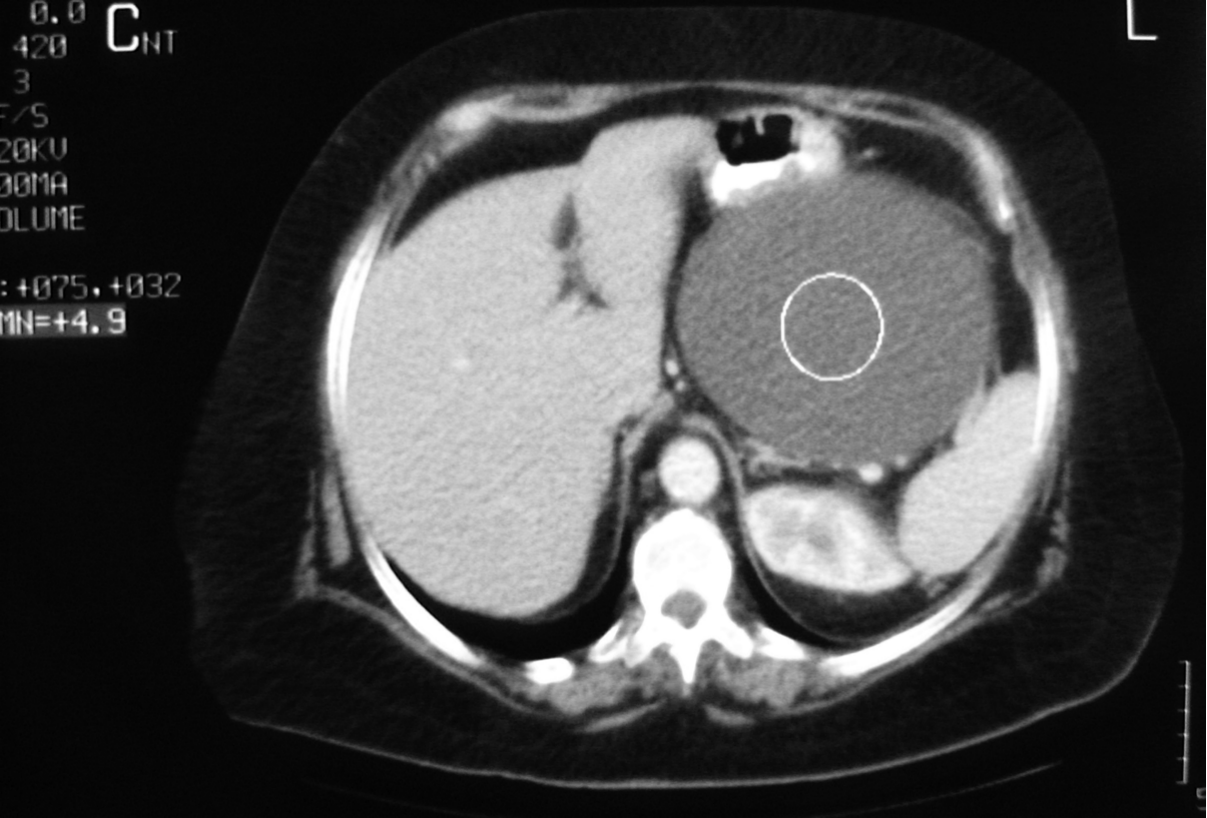
**CT examination with the Hounsfield (HU) value of the lesion demonstrating its cystic nature**.

Computed tomography (CT) examination demonstrated a large 18.7 × 13.2 × 22cm, intra-peritoneal hypodense mass in her upper left abdomen, lying between the great curvature of the stomach, the spleen and the tail of the pancreas, and extending caudally to the upper margin of the pelvis, causing pressure over the adjacent organs. The mass demonstrated no internal septa and no enhancement after the intravenous administration of a contrast medium. No abnormal lymphadenopathy was present (Figure 2, Figure 3 and Figure 4).

She underwent an urgent operation and the multicystic mass was found to occupy her entire left abdomen, adherent to the spleen. A complete resection of the lesion and splenectomy were performed. She had an uneventful post-operative recovery and a post-splenectomy prophylaxis was used.

**Figure 5:**
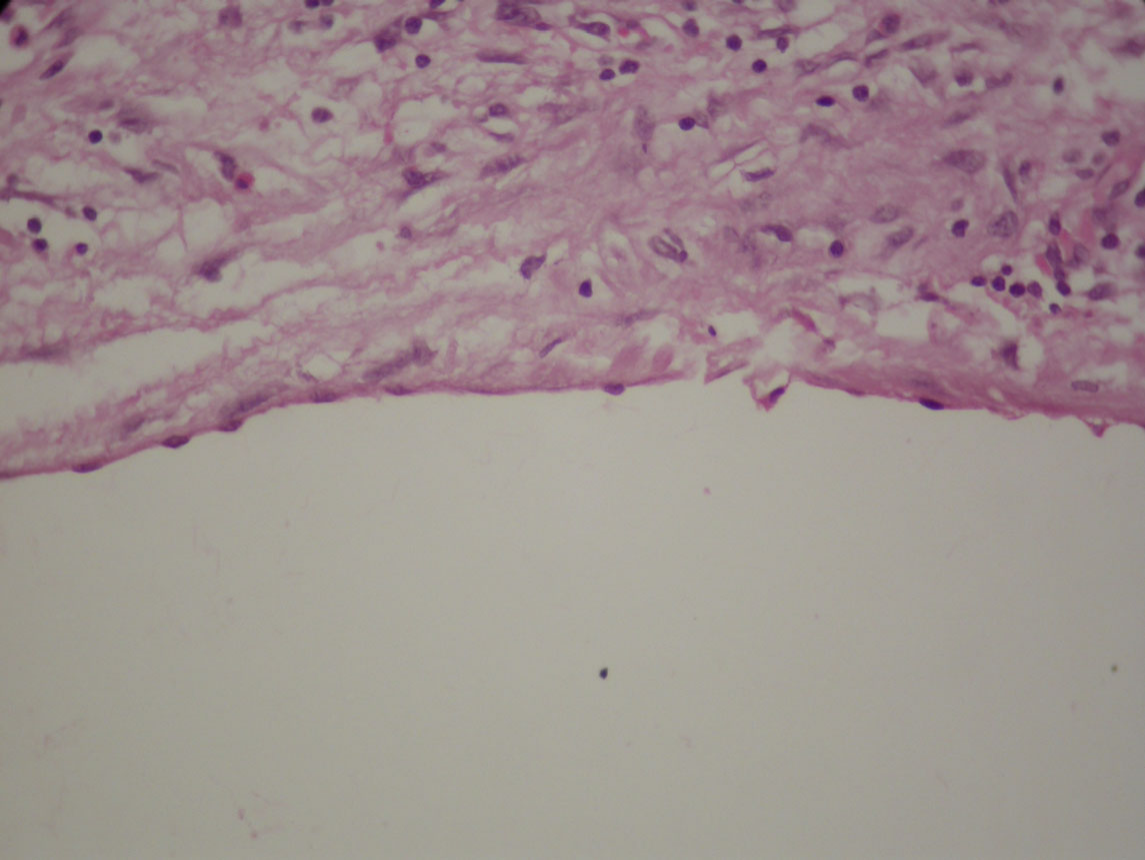
**Microscopic view of the benign multicystic peritoneal mesothelioma showing the mesothelial cells lining the cysts**. (Hematoxylin and eosin stain, original magnification × 400).

**Figure 6:**
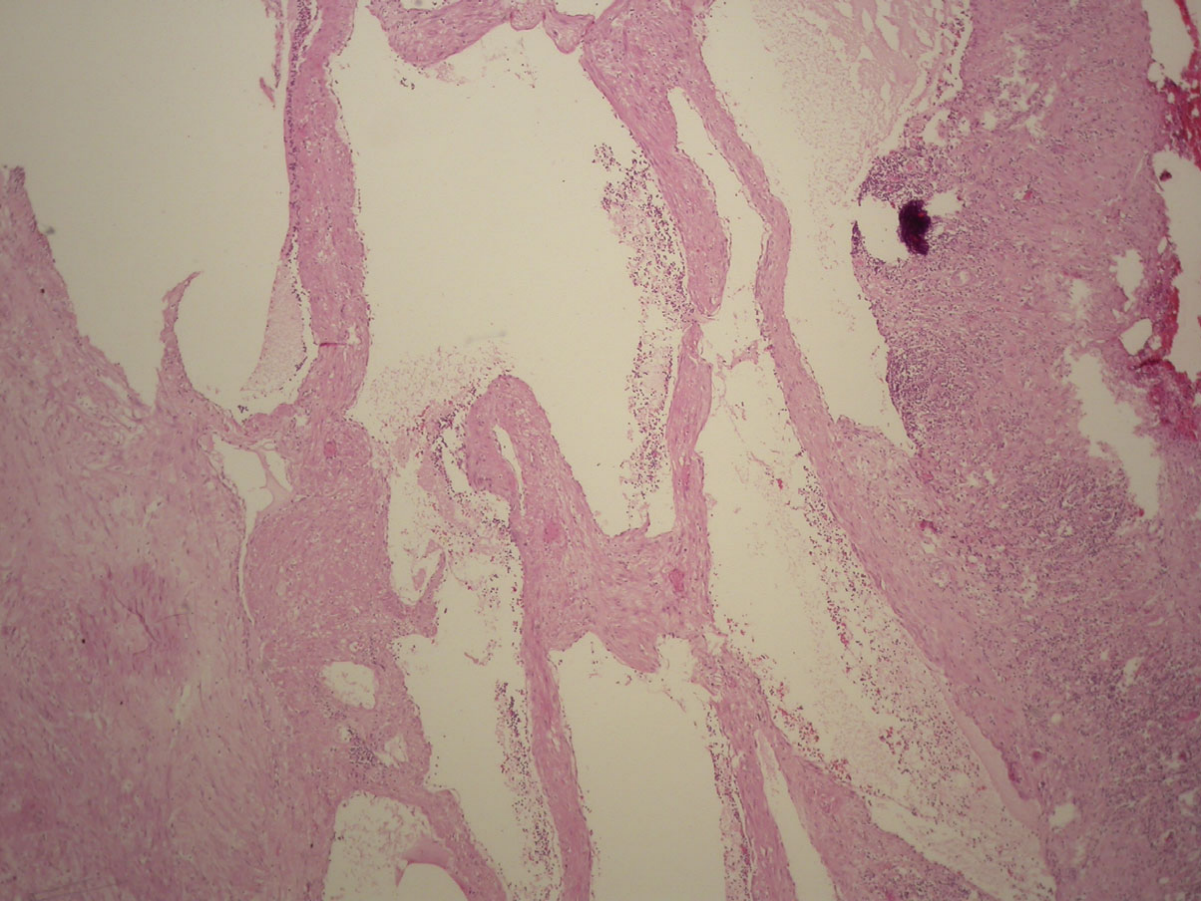
**Microscopic view showing the multicystic nature of the tumor**. (Hematoxylin and eosin stain, original magnification × 100).

Gross examination of the specimen showed a large gelatinous cystic mass containing multiple smaller cystic spaces. Her immunohistochemical stains were positive for calretinin and cytokeratins, confirming the mesothelial origin of the mass. The final diagnosis was benign multicystic peritoneal mesothelioma. (Figures 5 and 6).

Six months post-operatively, she had experienced no recurrence and was free of symptoms.

## Discussion

Mesotheliomas are mesenchymal neoplasms originating from the serous lining of the pleural, pericardial or peritoneal space. Multicystic peritoneal mesothelioma involves the peritoneum or extra-peritoneal space, omentum, pelvic or abdominal viscera. It most commonly arises from the pelvic surfaces of the peritoneum and has benign or indolent biologic behavior. Multicystic mesothelioma of the peritoneum was first described in 1979 by Mennemeyer and Smith and since then approximately 130 cases have been described in the literature. It is an intermediate-grade tumor, among the benign adenomatoid tumors of the peritoneum and the more common malignant asbestos-related peritoneal mesothelioma. It is not related to prior asbestos exposure and may recur locally [1-11].

On histological examination, the mesothelial cells lining the cysts may vary from flattened and endothelial-like to cuboidal. The thin-walled cysts may be filled with eosinophilic, serous fluid. Inflammatory cells and fibrous elements may be found within the stroma between the cysts. Foci of mesothelial hyperplasia may also be present [2].

It is usually large at the time of diagnosis (mean diameter, 13 cm). Multifocality, free floating cysts and unilocular cysts have been reported [2].

It commonly occurs in young to middle-aged women (mean age, 37 years). The presenting symptoms are chronic or intermittent lower abdominal or pelvic pain, tenderness, or distension with an abdominal or pelvic mass and, rarely, dyspareunia, constipation and urinary hesitancy and/or frequency. Women with this lesion often have a history of prior pelvic surgery, endometriosis or pelvic inflammatory disease [1-3,5,7-10].

The pathogenesis of benign multicystic peritoneal mesothelioma is unclear and there is some controversy regarding its neoplastic and reactive nature [2,6]. The fact that the great majority of patients are women of reproductive age suggests that a key role is played by female sex hormones in its pathogenesis [5].

US demonstrates multiseptated anechoic cysts. The fluid within the cysts is generally anechoic, but the cysts may contain echoes from debris or hemorrhage. The number and complexity of septations, as well as the size of the cysts, are quite variable. Calcification has not been described in multicystic mesothelioma. CT provides more information about the location and extent of the mass, and demonstrates a well-defined, low-attenuation mass with non-calcified septa. The septa become enhanced following intravenous administration of a contrast material. Magnetic resonance imaging (MRI) provides additional coronal and sagittal planes. The watery serous content has low signal intensity on T1-weighted images and intermediate-to-high signal intensity on T2-weighted images. Septal enhancement has been reported [1,2].

The differential diagnosis includes lymphangioma, other mesenteric and/or omental cysts, cystic teratoma, pseudomyxoma peritonei, cystic smooth muscle tumors, visceral cysts, cystic mucinous neoplasms of the pancreas, non-pancreatic pseudocysts, endometriosis, cystic adenomatoid tumor and cystic mesonephric duct remnants. When multicystic mesothelioma is located solely in the pelvis in women, tubo-ovarian abscess, hydrosalpinx, cystic ovarian neoplasms (ovarian cystadenoma, cystadenocarcinoma) and cystic forms of endosalpingiosis should be considered in the differential diagnosis. Lymphangiomas often occur in younger patients and can be identified if they contain predominantly chylous fluid and microscopically lymphoid aggregates and smooth muscle in their walls. Mesenteric cysts are generally unilocular and contain serous secretions, with no discernible wall or internal septa. Teratomas contain fat and calcification. Pseudomyxoma peritonei can be distinguished when there is co-existing omental caking, soft-tissue peritoneal nodules and scalloping of the serosal margins of the liver or spleen. The cystic component in cystic adenomatoid tumor is usually accompanied by a recognizable solid component. Malignant neoplasms are suggested by ancillary signs such as intramural nodules, ascites, necrosis or peritoneal carcinomatosis, and the source organ can usually be identified [1,5-7,9,10].

Multicystic mesothelioma is seldom diagnosed at pre-operative imaging because it is exceedingly rare; the diagnosis requires histological evaluation.

The treatment of choice is complete surgical excision. Complete removal of the cystic lesion, if possible, is the best treatment and the only hope in avoiding local recurrence. Aggressive surgical approaches including cytoreductive surgery with peritonectomy are recommended [5,6]. Hormonal therapy with anti-estrogens and gonadotrophin-releasing analogues, sclerotherapy with tetracycline, hyperthermic peritoneal perfusion with cisplatin and peritonectomy with intra-peritoneal chemotherapy have also been attempted in individual cases with varied degrees of success. Adjuvant chemotherapy and radiotherapy are not indicated as this tumor has a prevailing benign character [5,6].

About 50 percent of the patients experience a recurrence one to 27 years after the initial diagnosis and malignant transformation has very rarely been reported [1-3]. Thus, routine follow-up imaging is required post-operatively in all patients [4].

The prognosis is excellent and the only death that has ever been reported in the literature occurred in the case of a patient who refused to undergo resection 12 years after diagnosis [5].

## Conclusions

Benign multicystic peritoneal mesothelioma is a very rare benign cystic tumor. This lesion has a non-specific appearance on imaging which does not permit differential diagnosis from other cystic lesions and always requires histological evaluation. It has a high recurrence rate after surgical resection but malignant transformation has very rarely been reported. A systematic follow-up of these patients is required and further resection or other therapy may be indicated.

## Abbreviations

(CT): Computed tomography; (MRI): magnetic resonance imaging; (US): ultrasonography.

## Consent

Written informed consent was obtained from the patient for publication of this case report and any accompanying images. A copy of the written consent is available for review by the Editor-in-Chief of this journal.

## Competing interests

The authors declare that they have no competing interests.

## Authors' contributions

XP performed the chart review and prepared the manuscript. EA, AE and CT carried out the operation. LP was the pathologist who examined the specimen. AP, NS and TC participated in the preparation of the manuscript. All authors read and approved the final manuscript.
